# Transforming the canonical piecewise-linear model into a smooth-piecewise representation

**DOI:** 10.1186/s40064-016-3278-y

**Published:** 2016-09-20

**Authors:** Victor M. Jimenez-Fernandez, Maribel Jimenez-Fernandez, Hector Vazquez-Leal, Evodio Muñoz-Aguirre, Hector H. Cerecedo-Nuñez, Uriel A. Filobello-Niño, Francisco J. Castro-Gonzalez

**Affiliations:** 1 Faculty of Electronic Instrumentation, Universidad Veracruzana, Circuito Gonzalo Aguirre Beltrán S/N, Zona Universitaria, 91000 Xalapa, Veracruz Mexico; 2 Basic Sciences Institute, Universidad Veracruzana, Av. Castelazo Ayala S/N, Col. Industrial Ánimas, 91192 Xalapa, Veracruz Mexico; 3 Faculty of Mathematics, Universidad Veracruzana, Circuito Gonzalo Aguirre Beltrán S/N, Zona Universitaria, 91000 Xalapa, Veracruz Mexico; 4 Faculty of Physics, Universidad Veracruzana, Circuito Gonzalo Aguirre Beltrán S/N, Zona Universitaria, 91000 Xalapa, Veracruz Mexico

**Keywords:** Smooth, Canonical, Piecewise-linear, Model

## Abstract

A smoothed representation (based on natural exponential and logarithmic functions) for the canonical piecewise-linear model, is presented. The result is a completely differentiable formulation that exhibits interesting properties, like preserving the parameters of the original piecewise-linear model in such a way that they can be directly inherited to the smooth model in order to determine their parameters, the capability of controlling not only the smoothness grade, but also the approximation accuracy at specific breakpoint locations, a lower or equal overshooting for high order derivatives in comparison with other approaches, and the additional advantage of being expressed in a reduced mathematical form with only two types of inverse functions (logarithmic and exponential). By numerical simulation examples, this proposal is verified and well-illustrated.

## Background

Piecewise-linear models are widely used in diverse fields, such as circuit theory, image processing, system identification, economics and financial analysis, etc (Chua and Ying 1983; Chua and Deng 1985; Hasler and Schnetzler 1989; Yamamura and Ochiai 1992; Russo 2006; Feo and Storace 2004, 2007; Brooks 2008). The factor that prevalently motivates the use of this type of models is the simplicity of their structure which let them be efficiently implemented in both algorithms and hardware. In general, piecewise-linear models looks very appealing for graphical tasks, like curve fitting, interpolation or extrapolation, where a function is constructed to fit or determine new values within or outside the range of a discrete set of known data points (Bian and Menz 1998; Dai et al. 2007; Magnani and Boyd 2009; Misener and Floudas 2010; Jimenez-Fernandez et al. 2014). However, a notorious shortcoming can be distinguished in this type of models when function derivatives are of interest. This is because the first derivatives of piecewise-linear functions are not continuous at breakpoints and the second derivatives do not exist or are vanished inside each linear partition. This fact limits their application in that cases where derivatives are imperatively required, such as device modeling, nonlinear systems simulation, and analysis of experimental data, among others. In that regard, although there are many reported piecewise-linear models (Chua and Kang 1977; Kang and Chua 1978; Chua and Deng 1988; Kahlert and Chua 1990; Guzelis and Goknar 1991; Pospisil 1991; Kevenaar et al. 1994; Leenaerts and Van-Bokhoven 1998; Julian et al. 1999; Li et al. 2001), due to its compact formulation, the most popular is the so-called canonical piecewise-linear representation (Chua and Kang 1977) which is given by the following theorem:

### **Theorem 1**

*Any single*-*valued piecewise*-*linear function with at most*$\sigma$*breakpoints*$\beta_{1} < \beta_{2} < \ldots < \beta_{\sigma }$*, can be represented by the expression*

1 $$y(x) = a + bx + \sum\limits_{i = 1}^{\sigma } {c_{i} \left| {x - \beta_{i} } \right|}$$

*with*$$b = \left( {\frac{{J^{(1)} + J^{(\sigma + 1)} }}{2}} \right),$$$$c_{i} = \left( {\frac{{J^{(i + 1)} - J^{(i)} }}{2}} \right),$$$$a = y\left( 0 \right) - \sum\nolimits_{i = 1}^{\sigma } {c_{i} |\beta_{i} |}$$*for*$$i = 1,2, \ldots ,\sigma$$*, and*$$J^{\left( i \right)}$$*denoting the slope of the i*-*th constitutive linear segment in the piecewise*-*linear function.*

and more generally, for *n*-dimensional functions, (1) takes the form

2 $$y\left( {\mathbf{x}} \right) = a + {\mathbf{{\rm B}x}} + \sum\limits_{i = 1}^{\sigma } {c_{i} \left| {\left\langle {{\user2{\Lambda}}^{\left( i \right)} ,{\mathbf{x}}} \right\rangle - \beta_{i} } \right|}$$

where ${\mathbf{x}}$, ${\mathbf{\rm B}}$, and ${\user2{\Lambda}}^{\left( i \right)}$ are *n*-dimensional vectors, $a$, $c_{i}$ and $\beta_{i}$ are scalars, and “$\left\langle , \right\rangle$” denotes the inner product of two vectors.

As can be seen, this model is expressed by a closed formula with a minimal number of parameters. Nevertheless, due to a sum of absolute-value terms is included in (1) and (2), it is not completely differentiable.

Motivated by the fact of merging in a unique piecewise model these two fundamental characteristics: simplicity and differentiability, in this paper an algebraic transformation to smooth the canonical piecewise-linear model, is proposed. Such transformation let it obtain a new formulation which is based on natural exponential and logarithmic functions. It results in a new model that, besides of being smooth and preserving a minimum number of parameters, it makes the native piecewise-linear model completely differentiable. In this concern, it is important to mention that, in accordance with literature such lack of differentiability has been overcome by substituting the basis-function of the piecewise-linear model (in this case, the absolute-value) for its smooth approximation. Illustrative examples of this strategy can be found in (Bacon and Watts 1971; Seber and Wild 1989; Lazaro et al. 2001; Griffiths and Miller 1973), where the functions $sign(x)$, $\tanh (x)$, $lch(x)$, and $hyp(x)$ are used, respectively. Similarly, our smoothing transformation is based on the same principle but compared to those reported approaches, it reveals significant improvements, for example: (1) a better curve fitting accuracy can be achieved due to the deviation between the piecewise-linear function of reference, and the resulting smooth description, is restrictively focused at the breakpoints, (2) no additional parameters needs to be computed because it uses the same parameters of the original canonical model, and (3) the resulting smooth model exhibits a lower or equal overshooting for their derivatives. The paper is organized as follows. In section 2, the deduction of the smoothing transformation formula is explained in detail. Section 3 describes the transformation strategy by two illustrative examples (for one- and two-dimensional domains). In section 4 a comparative analysis and discussion about the curve fitting accuracy that can be achieved through the proposed transformation as well as the overshooting in derivatives, is exposed. This comparative is done among the most popular smoothing proposals reported in literature. Finally, section 5 presents the concluding remarks of this work.

## Deduction of transformation formula

It follows from (Schmidt et al. 2007) that a smooth approximation for the absolute-value function can be expressed in terms of natural logarithms as

3 $$\left| x \right| = \frac{1}{{\alpha \ln \left( {10} \right)}}\left[ {\ln \left( {1 + e^{ - \alpha x} } \right) + \ln \left( {1 + e^{{\left( {\alpha x} \right)}} } \right)} \right]$$

Using the property $\ln \left( {uv} \right) = \ln \left( u \right) + \ln \left( v \right)$ in (3), and simplifying the resulting algebraic expression we have

4 $$\left| x \right| = \frac{2}{{\alpha \ln \left( {10} \right)}}\ln \left( {e^{{\left( {\frac{\alpha }{2}x} \right)}} + e^{{\left( {\frac{ - \alpha }{2}x} \right)}} } \right)$$

After numerical simulations on (4), a slight deviation from the unity slope of the absolute-value function can be observed. In order to achieve more approximation accuracy, a constant *µ* is included as

5 $$\left| x \right| = \frac{2}{{\alpha \ln \left( {10} \right)}}\mu \ln \left( {e^{{\left( {\frac{\alpha }{2}x} \right)}} + e^{{\left( {\frac{ - \alpha }{2}x} \right)}} } \right)$$

being $\mu = \ln \left( {10} \right)$ an appropriate fitting value.

This simplify (5) as

6 $$\left| x \right| = \frac{2}{\alpha }\ln \left( {e^{{\left( {\frac{\alpha }{2}x} \right)}} + e^{{\left( {\frac{ - \alpha }{2}x} \right)}} } \right)$$

*Proof* See Appendix A

After substituting the absolute-value function of (1) by its equivalent smooth approximation (6), it let us recast the canonical model as

7 $$y\left( x \right) = a + bx + \sum\limits_{i = 1}^{\sigma } {c_{i} \left( {\frac{2}{\alpha }} \right)\ln \left( {e^{{\frac{\alpha }{2}\left( {x - \beta_{i} } \right)}} + e^{{\frac{ - \alpha }{2}\left( {x - \beta_{i} } \right)}} } \right)}$$

Hence, performing an algebraic reduction of (7) yields:

8 $$y\left( x \right) = a + bx + \sum\limits_{i = 1}^{\sigma } {c_{i} \left( {x - \beta_{i} } \right) + \frac{2}{\alpha }\sum\limits_{i = 1}^{\sigma } {c_{i} \ln \left( {1 + e^{{ - \alpha \left( {x - \beta_{i} } \right)}} } \right)} }$$

that hereafter is denoted as the smooth-piecewise model whose parameters ($a$,$b$, and $c_{i}$) are the same as the canonical piecewise-linear model, and the parameter *α* is incorporated to controls the smoothness. A more formal definition for (8) is expressed by the following theorem:

### **Theorem 2**

*Any one*-*dimensional canonical piecewise*-*linear function that is characterized by L segments and σ breakpoints*$\beta_{1} < \beta_{2} < \ldots < \beta_{\sigma } ,$*can be transformed into smooth*-*piecewise function expressed as*

9 $$y\left( x \right) = A + Bx + \sum\limits_{i = 1}^{\sigma } {C_{i} \ln \left( {1 + e^{{ - \alpha \left( {x - \beta_{i} } \right)}} } \right)} \quad with\,\sigma = (L - 1)$$

*where the set of*$\left( {\sigma + 2} \right)$*parameters:*$\left\{ {A,B,C_{i} } \right\}$*can be determined as follows:*

10 $$A = a - \sum\limits_{i = 1}^{\sigma } {c_{i} \beta_{i} }$$

11 $$B = b + \sum\limits_{i = 1}^{\sigma } {c_{i} }$$

12 $$C_{i} = \frac{{2c_{i} }}{\alpha }\quad for\,i = 1 \ldots \sigma$$

*and the parameter α can be used to preserve a constant smoothness in all the function domain, or to define a specific smoothness grade*$\alpha_{i}$*at any i*-*th breakpoint location as*

13 $$\alpha_{i} = \frac{{2c_{i} \ln \left( 2 \right)}}{\delta }$$

with *δ* being the deviation between the piecewise-linear and the smooth-piecewise functions at $x = \beta_{i} .$

*Proof* See Appendix B

Without loss of generality, for an *n*-dimensional representation of (9), a smooth transformation is derived from (2) and expressed as

14 $$ y\left( {\mathbf{x}} \right) = A + {{\hat{\mathbf{\rm B}}x}} \, + \sum\limits_{i = 1}^{\sigma } {C_{i} \ln \left( {1 + e^{{ - \alpha \left( {\left\langle {{\user2{\Lambda}}^{\left( i \right)} ,{\mathbf{x}}} \right\rangle - \beta_{i} } \right)}} } \right)}$$

where both parameters, $A$ and $C_{i}$, are calculated by using the same equations as for the one-dimensional case (Eqs. (10), (12), respectively), and ${\hat{\rm B}}$ is determined as follows:

15 $${{\hat{\rm B}}} = {\mathbf{\rm B}} + \sum\limits_{i = 1}^{\sigma } {c_{i} {\user2{\Lambda}}^{(i)} }$$

*Proof* See Appendix C

## Illustrative examples

With the purpose of exploring (9), we present two application examples; the first shows how to obtain the smooth-piecewise representation of any one-dimensional function, and the second exposes a more practical case where the smoothing transformation is applied into a two-dimensional characterization curve for a n-channel MOS transistor.

### Example 1

Consider any one-dimensional piecewise-linear function $y_{PWL} \left( x \right)$ characterized by the following linear segments:

16 $$y_{PWL} \left( x \right) = \left\{ {\begin{array}{*{20}c} {\frac{1}{2}x} &\quad { - \infty < x < 1} \\ { - x + \frac{3}{2}} &\quad {1 < x < 2} \\ {\frac{3}{2}x - \frac{7}{2}} &\quad {2 < x < 3} \\ { - x + 4} &\quad {3 < x < 4} \\ {x - 4} &\quad {4 < x < + \infty } \\ \end{array} } \right.$$

from (16), it can be directly obtained: $$L = 5,$$$$\sigma = 4,$$$$\beta = \left\{ {1,2,3,4} \right\},$$ and $$J = \left\{ { + \frac{1}{2}, - 1, + \frac{3}{2}, - 1, + 1} \right\}.$$

After substituting the slopes ($J^{\left( i \right)}$ for $i = 1,2, \ldots ,5$) and breakpoints ($\beta_{i}$ for $i = 1,2, \ldots ,4$) values, into the parameters of (1) and substituting in (10), (11), and (12), the smooth description (17) can be obtained.

17 $$\begin{aligned} y\left( x \right) &= - 4 + x + \frac{2}{\alpha }\left( { - \frac{3}{4}\ln \left( {1 + e^{{ - \alpha \left( {x - 1} \right)}} } \right)} \right) + \frac{2}{\alpha }\left( {\frac{5}{4}\ln \left( {1 + e^{{ - \alpha \left( {x - 2} \right)}} } \right)} \right) \\ & \quad - \frac{2}{\alpha }\left( {\frac{5}{4}\ln \left( {1 + e^{{ - \alpha \left( {x - 3} \right)}} } \right)} \right) + \frac{2}{\alpha }\left( {\ln \left( {1 + e^{{ - \alpha \left( {x - 4} \right)}} } \right)} \right) \end{aligned}$$

**Fig. 1:**
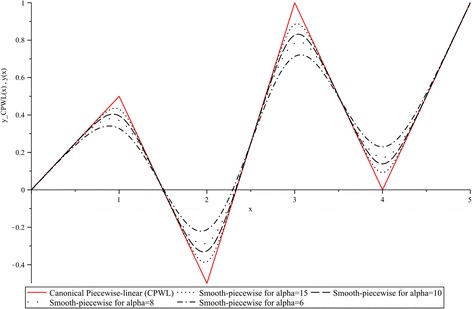
Smooth-piecewise approximations of $y_{CPWL} (x)$ for $\alpha = \left\{ {6,8,10,15} \right\}$

In order to exemplify how the smoothness can be controlled by fixing the value of the parameter $\alpha$, a summary of curves (black) for $\alpha = \left\{ {6,8,10,15} \right\}$ is reported in Fig. 1. As reference, the original piecewise-linear curve $y_{CPWL} \left( x \right)$, derived from Theorem 1, is also included in this figure (red).

18 $$y_{CPWL} \left( x \right) = - 2 + \frac{3}{4}x - \frac{3}{4}\left| {x - 1} \right| + \frac{5}{4}\left| {x - 2} \right| - \frac{5}{4}\left| {x - 3} \right| + \left| {x - 4} \right|$$

**Fig. 2:**
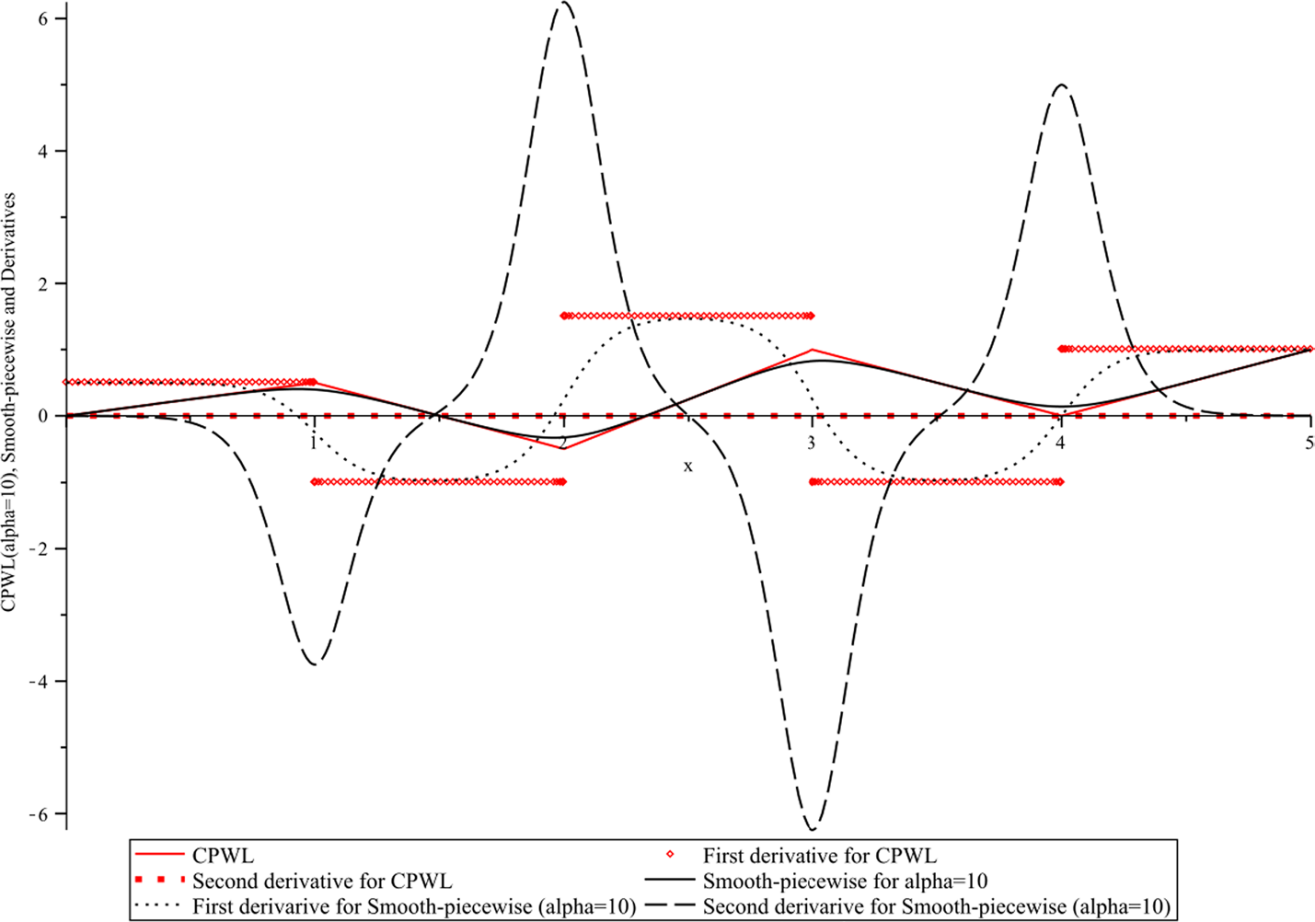
First and second derivatives for $y_{CPWL} (x)$ and $y(x),$ ($\alpha = 10$)

From this figure it can be observed that, when the parameter *α* is small the smoothness of (17) is increased, in contrast, when *α* is greater it is decreased. From a geometrical interpretation, this means a trade-off between the deviation from the breakpoint coordinate, and the desired smoothness. In Fig. 2 the first and second derivatives of $y(x),$ for $\alpha = 10,$ are contrasted with the corresponding derivatives of $y_{CPWL} (x).$ As it was expected, the first derivative for $y_{CPWL} (x)$ yields a discontinuous step curve, and the second and higher order derivatives are always zero. In contrast, it must be highlighted the existence of the first and higher order derivatives for the smooth function.

### Example 2

In order to illustrate the smoothing transformation for a two-dimensional function, the characteristic curves and equilibrium equations of a metal–oxide–semiconductor (MOS) field-effect transistor are considered. This is a four-terminal device: source (S), gate (G), drain (D), and body (B) which is used for amplifying or switching electronic signals.

Let us start considering a n-channel MOS transistor connected in the common source configuration with $$v_{1} = v_{GS} ,$$$$v_{2} = v_{DS} ,$$ and $$i_{2} = i_{D} ,$$ where $$v_{1}$$, $$v_{2}$$ are in volts, and $$i_{2}$$ are in microamperes. We assume that the piecewise-linear description $$i_{2}$$ follows the Shichman-Hodges model for $$k = 50\,\mu A/V^{2} ,$$$$V_{t} = 1\,V,$$$$\lambda = 0.02\,V^{ - 1} ,$$ and it is expressed in the canonical form of the Chua model (Chua and Deng 1986) as follows:

19 $$\begin{aligned}i_{2,cpwl} &= - 12.405 + 3.286v_{1} + 71.493v_{2} + 0.438\left| {37.738v_{1} - v_{2} + 42.459} \right| \\ & \quad - 54.407\left| {0.6705v_{1} - v_{2} - 1.5385} \right| - 15.715\left| {1.043v_{1} - v_{2} - 1.3058} \right| \\ & \quad + 1.809\left| { - 21.904v_{1} - v_{2} + 54.166} \right| \end{aligned}$$

**Fig. 3:**
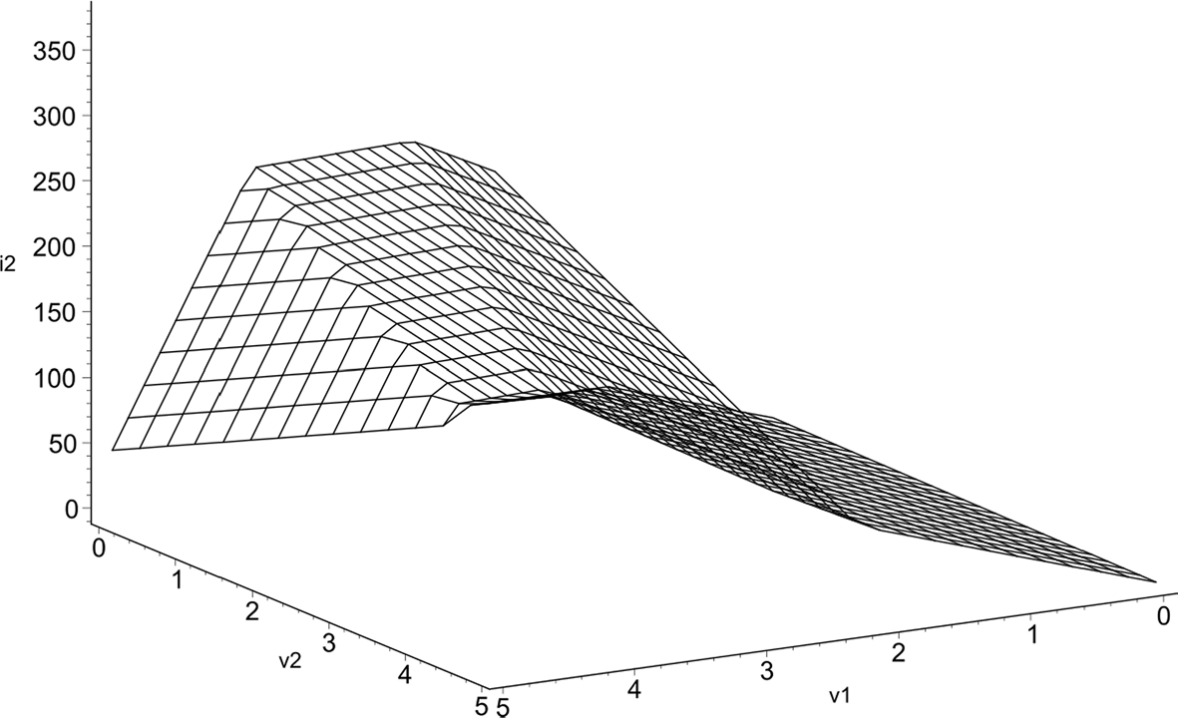
Canonical piecewise-linear approximation for the drain current $i_{2}$

The resulting piecewise-linear characteristic $y_{CPWL} \left( {v_{1} ,v_{2} } \right) = i_{2}$ is shown in Fig. 3.

From (19) in reference to (2) is obtained:

$$A = - 12.405,$$$${\mathbf{\rm B}} = \left[ {\begin{array}{*{20}c} {3.286} \\ {71.493} \\ \end{array} } \right],$$$${\user2{\Lambda}}^{\left( 1 \right)} = \left[ {\begin{array}{*{20}c} {37.738} \\ { - 1} \\ \end{array} } \right],$$$${\user2{\Lambda}}^{\left( 2 \right)} = \left[ {\begin{array}{*{20}c} {0.6705} \\ { - 1} \\ \end{array} } \right],$$$${\user2{\Lambda}}^{\left( 3 \right)} = \left[ {\begin{array}{*{20}c} {1.403} \\ { - 1} \\ \end{array} } \right],$$$${\user2{\Lambda}}^{\left( 4 \right)} = \left[ {\begin{array}{*{20}c} { - 21.904} \\ { - 1} \\ \end{array} } \right],$$$$c_{1} = 0.438,$$$$c_{2} = - 54.407,$$$$c_{3} = - 15.715,$$$$c_{4} = 1.809,$$$$\beta_{1} = - 42.459,$$$$\beta_{2} = 1.5385,$$$$\beta_{3} = 1.3058,$$$$\beta_{4} = - 54.166$$

After applying (10), (12) and (15) for $\alpha = 5$, it results

20 $$\begin{aligned}i_{2s} &= - 233.2142 + 79.2517v_{1} + 3.618v_{2} \\ & \quad + 0.1752\ln \left( {1 + e^{{188.69v_{1} - 5v_{2} + 212.295}} } \right) - 21.7628\ln \left( {1 + e^{{3.3525v_{1} - 5v_{2} - 7.6925}} } \right) \\ & \quad - 6.286\ln \left( {1 + e^{{5.215v_{1} - 5v_{2} - 6.529}} } \right) + 0.7236\ln \left( {1 + e^{{ - 109.52v_{1} - 5v_{2} + 270.83}} } \right) \end{aligned}$$

**Fig. 4:**
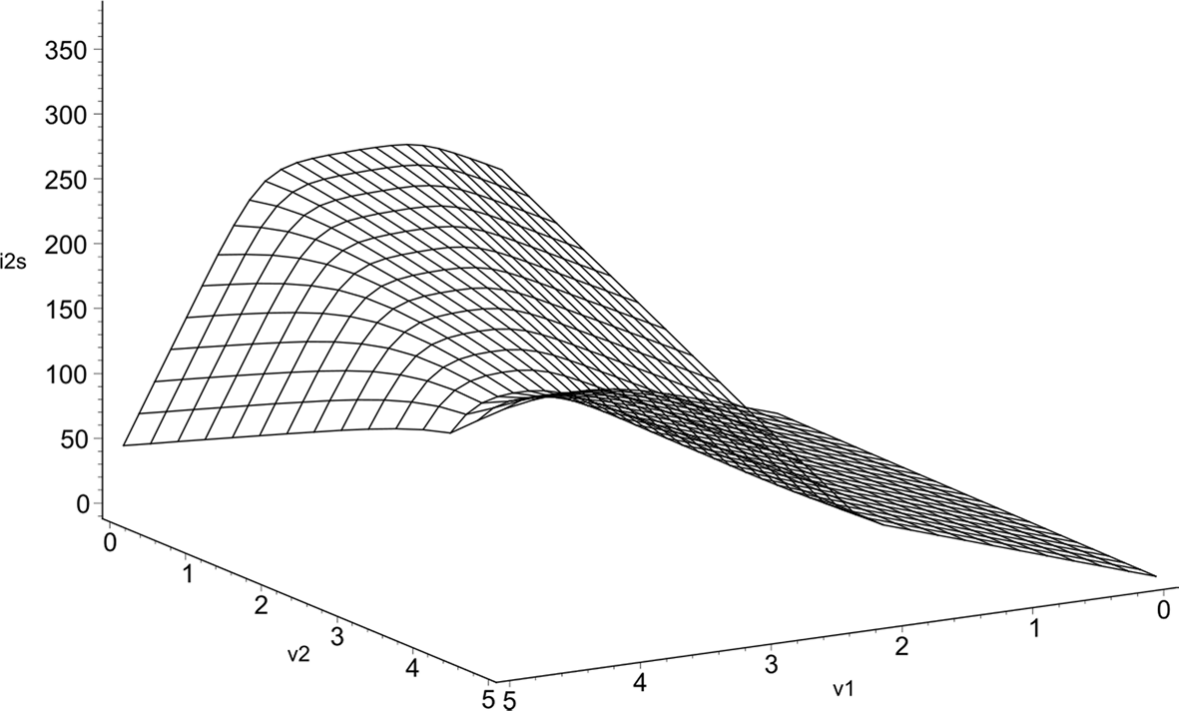
Smooth curve $i_{2s}$ that result from the transformation of $i_{2}$

Figure 4 shows the characteristic curve of $i_{2s} .$

**Fig. 5:**
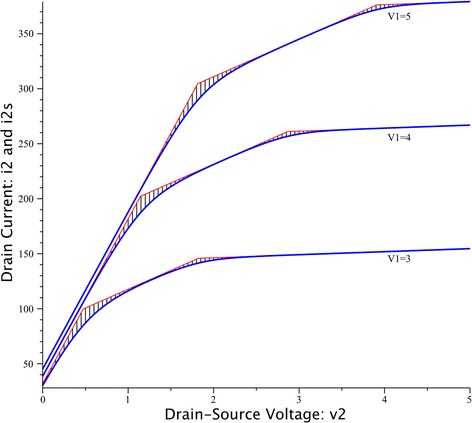
Deviation between the piecewise-linear function $i_{2}$ (*red*) and the smooth-piecewise function $i_{2s}$ (*blue*)

With the aim of estimating the difference between the piecewise-linear function $i_{2}$ and the smooth-piecewise function $i_{2s}$, the deviation between their characteristic curves is depicted in Fig. 5 (shadow regions). Similarly, as it happens in the one-dimensional example, the most precise curve fitting is achieved within each linear segment (in the two-dimensional case, each plane) but the deviation (controlled by the smoothing parameter *α*) only appears near the breakpoints.

## Comparative analysis and discussion

In this section, an analysis and discussion about the curve fitting accuracy and the overshooting in function derivatives due to the smooth-piecewise model (9), is outlined. In order to have a comparative reference, besides of our proposal, other smoothing alternatives are considered and illustrated.

### Smooth approximation for the absolute value function

As it was exposed in section 2, the proposed smoothing strategy uses an approximation for the absolute-value function based on a natural logarithmic with a Euler’s exponential argument, for simplicity, such approximation hereafter will be denoted as $lne$ and it is obtained by recasting (6) as follows

$$\frac{2}{\alpha }\ln \left( {e^{{\frac{\alpha }{2}x}} + e^{{\frac{ - \alpha }{2}x}} } \right) = \frac{2}{\alpha }\ln \left( {\frac{{e^{\alpha x} + 1}}{{e^{{\frac{\alpha }{2}x}} }}} \right) = \frac{2}{\alpha }\ln \left( {e^{\alpha x + 1} } \right) - \frac{2}{\alpha }\ln \left( {e^{{\frac{\alpha }{2}x}} } \right)$$

After simplifying, it results

21 $$lne = k_{1} \ln \left( {e^{{\frac{2x}{{k_{1} }}}} + 1} \right) - x$$

with $$k_{1} = \frac{2}{\alpha }$$

Other reported approximations are, the here denoted, $lch$ (Lazaro et al. 2001) and $th$ (Seber and Wild 1989). The first one based on the natural logarithm of a hyperbolic cosine argument, and the second one directly expressed in terms of a hyperbolic tangent. Both approximations are expressed as

22 $$lch = k_{2} \ln \left( {\cosh \left( {\frac{x}{{k_{2} }}} \right)} \right)$$

23 $$th = x\tanh \left( {k_{3} x} \right)$$

**Fig. 6:**
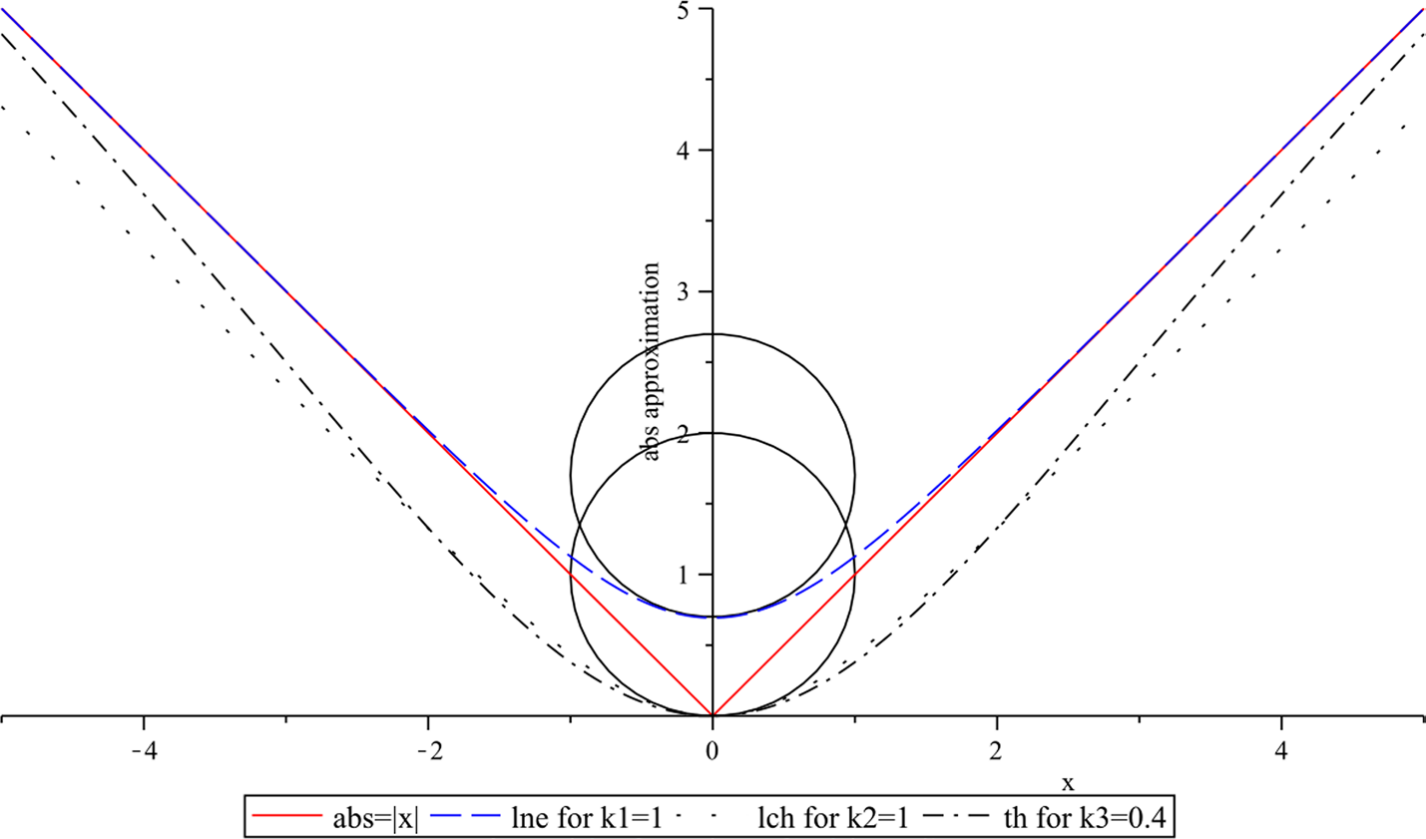
Smooth approximations for ${\text{abs}} = \left| x \right|$: $lne$ for $k_{1} = 1$, $lch$ for $k_{2} = 1$, and $th$ for $k_{3} = 0.4$

Figure 6 shows the absolute-value function $$abs = \left| x \right|$$ and their approximations $$lne,$$$$lch,$$ and $$th.$$ In all cases, the same curvature (smoothness) is considered ($$k_{1} = 1$$, $$k_{2} = 1,$$ and $$k_{3} = 0.4$$). This fact can be graphically observed by the circles that are circumscribed at the breakpoint.

**Fig. 7:**
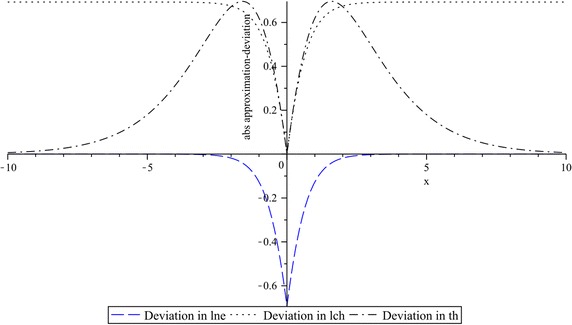
Curve fitting deviations in $lne$, $lch$ and $th$, with respect to the absolute-value function. $k_{1} = 1$, $k_{2} = 1$, and $k_{3} = 0.4$

For this example, curve fitting deviations of $lne$, $lch$, and $th$, with respect to the absolute-value function, are shown in Fig. 7 where clearly it can be seen that, in $lch$ and $th$, a considerable variation is presented, especially far away or in the two quadrants near the breakpoint at $x = 0$. However, in the close proximity of this point the curve deviation is progressively minimized. In contrast, by $lne$, the reciprocal behavior can be observed. That is to say, the main deviation is focused at $x = 0$, and it drops to zero as the curve moves away this point.

### Overshooting in derivatives of the absolute value function approximations

**Fig. 8:**
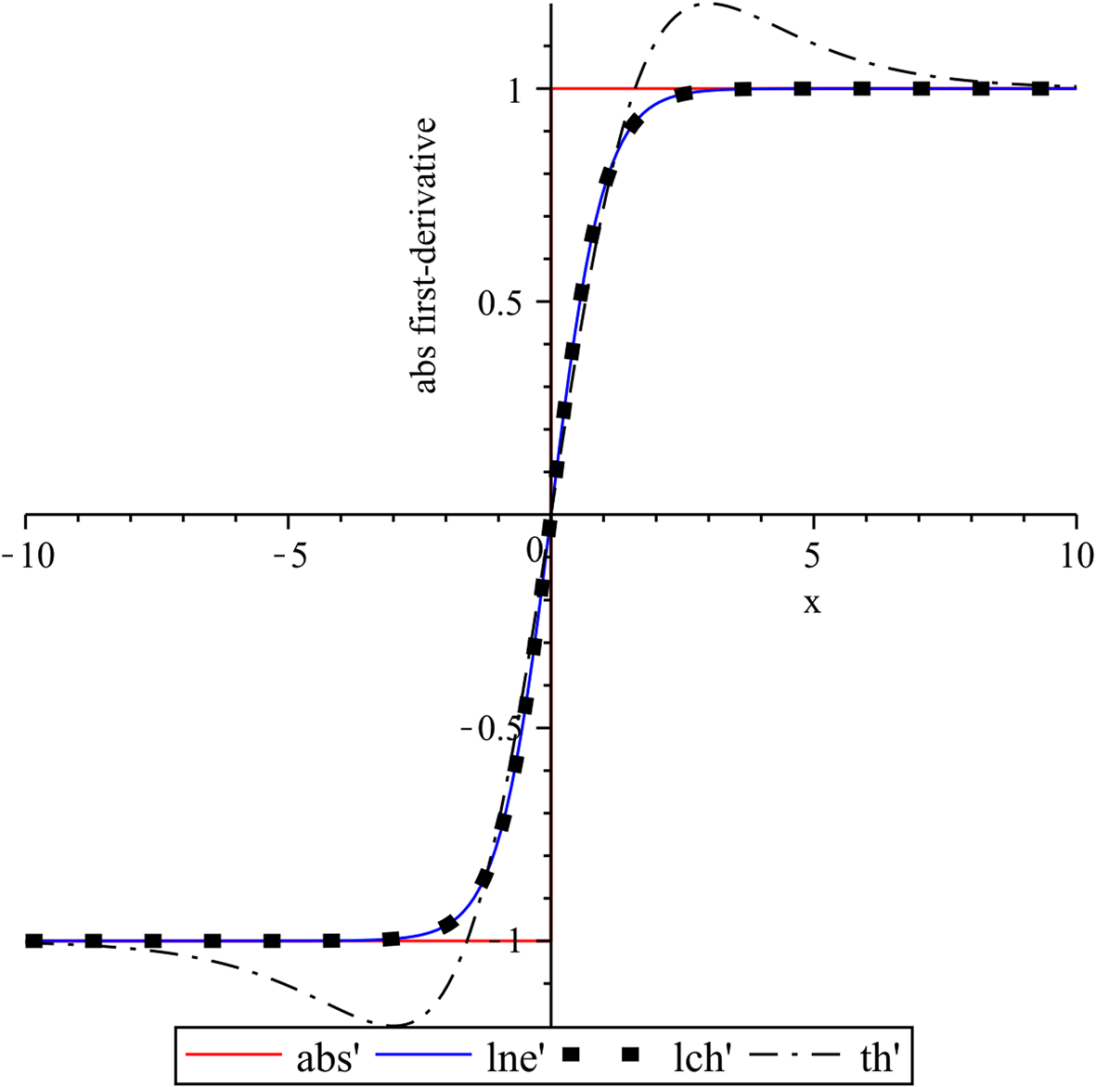
First derivatives: $lne^{\prime}$ of $lne$ for $k_{1} = 1$, $lch^{\prime}$ of $lch$ for $k_{2} = 1$, and $th^{\prime}$ of $th$ for $k_{3} = 0.4$

**Fig. 9:**
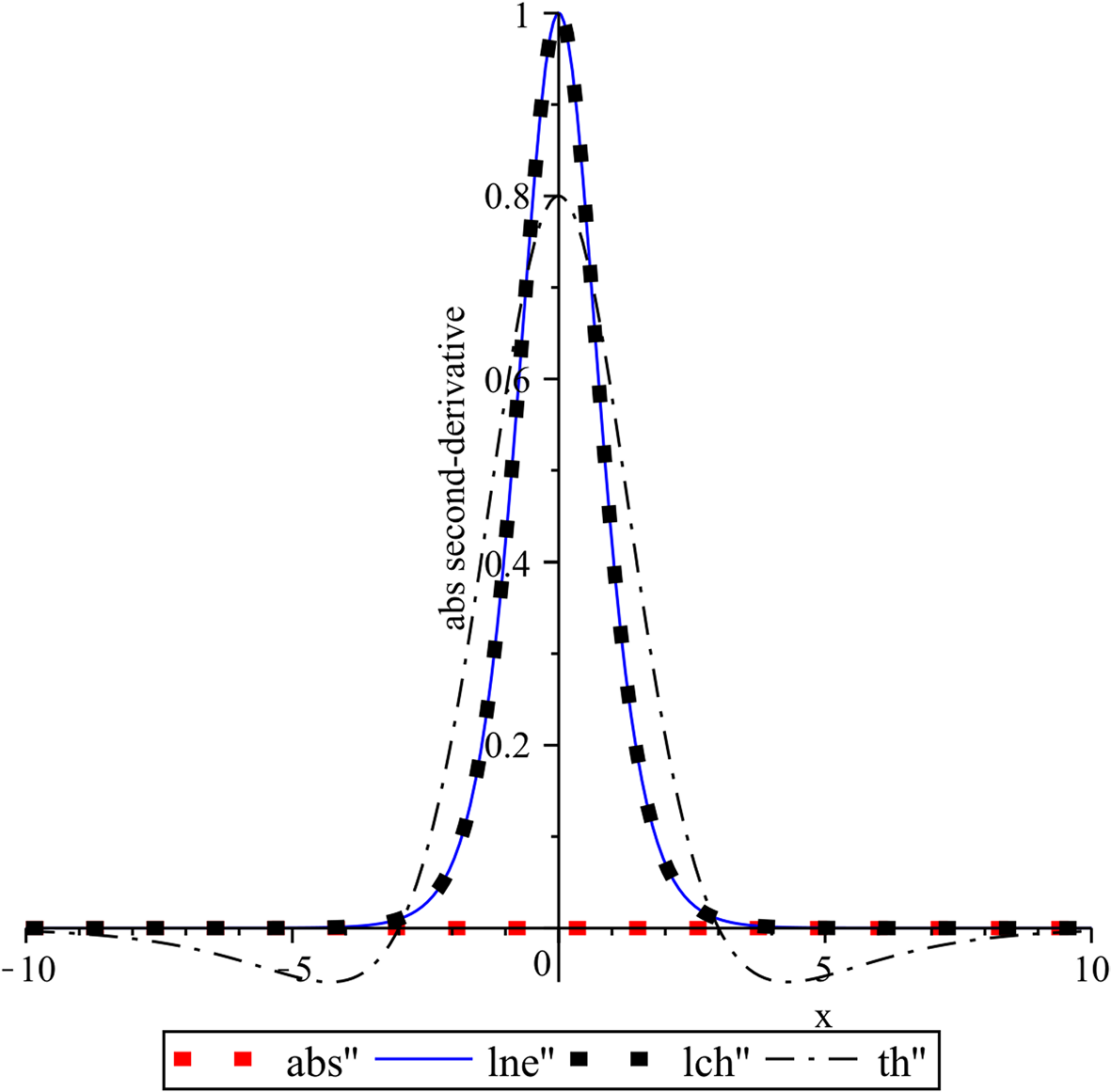
Second derivatives: $lne^{\prime\prime}$ of $lne$ for $k_{1} = 1$, $lch^{\prime\prime}$ of $lch$ for $k_{2} = 1$, and $th^{\prime\prime}$ of $th$ for $k_{3} = 0.4$

In Figs. 8 and 9 the first and second derivatives of functions: $lne$ for $k_{1} = 1$, $lch$ for $k_{2} = 1$, and $th$ for $k_{3} = 0.4$ are contrasted with the corresponding derivative of absolute-value function. As it was expected, the first derivative for the absolute-value function yields a step function while their second and higher order derivatives are always zero. We can also see that, in the both cases shown in Figs. 8 and 9, derivatives of $th$ exhibit more overshooting than $lne$ and $lch$. Moreover, it can be noted the same overshooting for $lne$ and $lch$.

### Comparative example

By this example, the two previously discussed characteristics: curve fitting of breakpoints and overshooting for function derivatives, are explored. Hence, consider a piecewise-linear curve defined by two breakpoints: $\beta = \left\{ {1,2} \right\}$, and three slopes: $J = \left\{ {2, - 3,1} \right\}$. In accordance with (1), from these input data the canonical piecewise-linear model description is given by

24 $$y_{pwl} \left( x \right) = - \frac{3}{2} + \frac{3}{2}x - \frac{5}{2}\left| {x - 1} \right| + 2\left| {x - 2} \right|$$

**Fig. 10:**
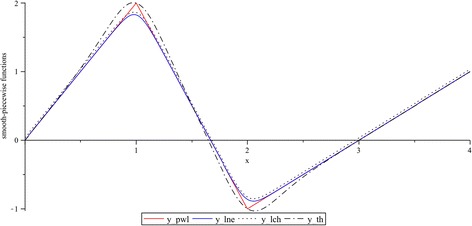
Smooth-piecewise transformation for $y_{pwl} \left( x \right)$. Functions: $y_{lne} \left( x \right)$, $y_{lch} \left( x \right)$ and $y_{th} \left( x \right)$ for $k_{1} = 0.1$, $k_{2} = 10$, and $k_{3} = 4$, respectively

Smoothing transformations of (24) can be now intuitively achieved by replacing the absolute-value function with any of their approximations ($lne$, $lch$, and $th$). After applying the corresponding substitutions, we obtain

25 $$y_{\ln e} (x) = 2x - \frac{1}{4}\ln (e^{20x - 20} + 1) + \frac{1}{5}\ln \left( {e^{20x - 40} + 1} \right)$$

26 $$y_{lch} (x) = - \frac{3}{2} + \frac{3}{2}x - \frac{1}{4}\ln \left( {\cosh \left( {10x - 10} \right)} \right) + \frac{1}{5}\ln \left( {\cosh \left( {10x - 20} \right)} \right)$$

27 $$y_{th} (x) = - \frac{3}{2} + \frac{3}{2}x - \frac{5}{2}\left( {x - 1} \right)\tanh \left( {4x - 4} \right) + 2\left( {x - 2} \right)\tanh \left( {4x - 8} \right)$$

where (25), (26), and (27), are the smooth functions that correspond to $lne$, $lch$, and $th$, respectively. It is important to point that, in order to evaluate these functions under equally conditions, the same smoothness is fixed by the parameters $k_{1} = 0.1$, $k_{2} = 10$, and $k_{3} = 4$. Plots for these functions are depicted in Fig. 10.

**Fig. 11:**
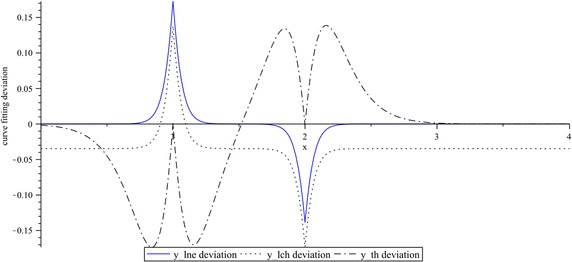
Curve fitting deviations of $lne$, $lch$ and $th$, with respect to the absolute-value function. $k_{1} = 0.1$, $k_{2} = 10$, and $k_{3} = 4$

Curve fitting deviations of (25), (26), and (27), with respect to the reference function (24) can be appreciated in Fig. 11. From this figure it can be seen that a minimum deviation corresponds to (25) and the worst case is given by (27).

**Fig. 12:**
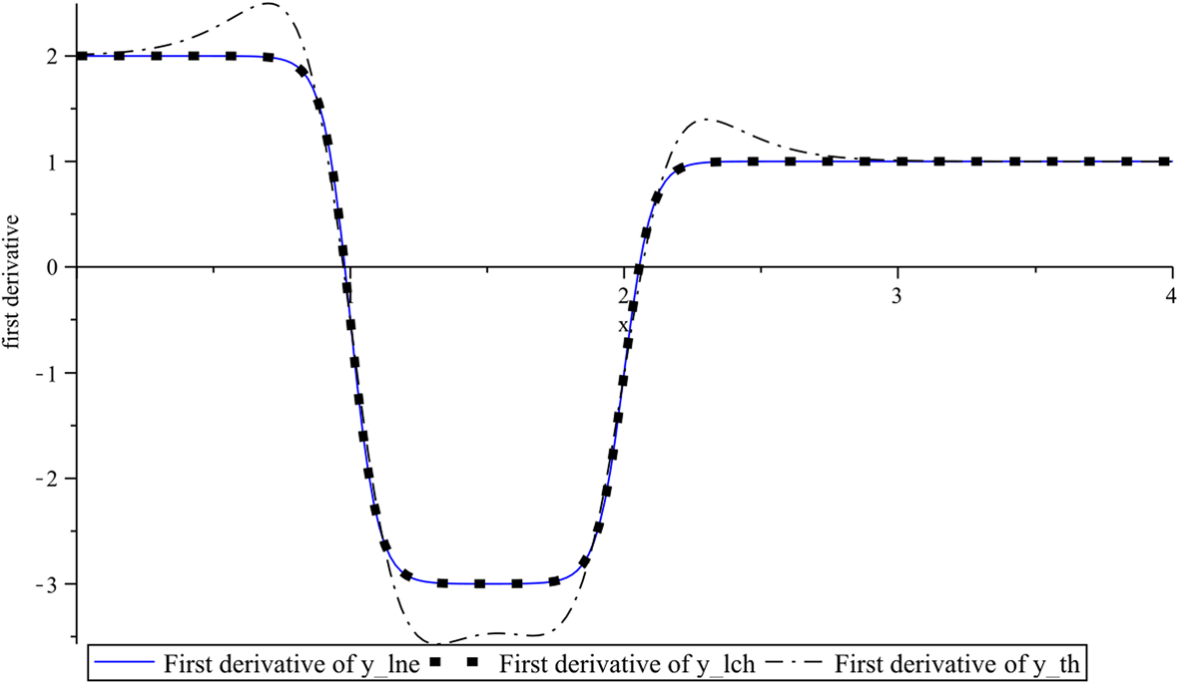
Overshooting for the first derivatives of $y_{lne} \left( x \right)$, $y_{lch} \left( x \right)$ and $y_{th} \left( x \right)$

Figure 12 shows that similarly as it was observed in Figs. 8 and 9, the most pronounced overshooting is due to the $th$ approximation while an equally overshooting corresponds to $lne$ and $lch$.

## Conclusion

The proposed transformation was successfully applied to one-dimensional and two-dimensional piecewise-linear functions. By numerical simulations, it was verified that in comparison with other reported strategies, our smooth-piecewise model has important advantages, like preserving the original parameters of its native canonical piecewise-linear representation, the capability of controlling the smoothness by an artificial parameter (*α*), a lower or equal overshooting for derivatives, and the additional advantage of being expressed in a more reduced mathematical form with only two types of inverse functions (logarithmic and exponential).

## Figures and Tables

**Fig. 13:**
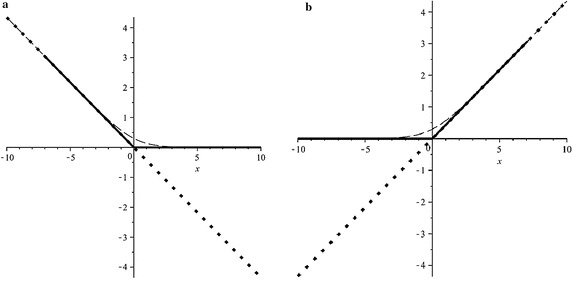
Graphs of (29) for $\alpha = 1$ (*dashed*), $\alpha = 100$ (*solid*) and their linear approximations (*dot*). **a** For $x$ negative, **b** for $x$ positive

## Appendix A

Let Eq. (3) be rewritten as

28 $$\left| x \right| \cong h\left( { - x,\alpha } \right) + h\left( {x,\alpha } \right)$$

where

29 $$h\left( {x,\alpha } \right) = \frac{1}{{\alpha \ln \left( {10} \right)}}\ln \left( {1 + e^{\alpha x} } \right)$$

In Fig. 13, the graphs of $h(x,\alpha )$ and $h( - x,\alpha )$ for low and high values of the smoothing parameter $\alpha$ (i.e. *α* = 1 and *α* = 100) are depicted in dashed and solid line styles, respectively. Their linear approximations are also traced in dot style.

From the above graphs, the following linear approximations are proposed.

30 $$h( - x,\alpha ) = \left\{ {\begin{array}{*{20}c} { - mx} & {\begin{array}{*{20}c} {\text{if}} & {x \le 0} \\ \end{array} } \\ 0 & {\begin{array}{*{20}c} {\text{if}} & {x > 0} \\ \end{array} } \\ \end{array} } \right. \quad h( x,\alpha ) = \left\{ {\begin{array}{*{20}c} { + mx} & {\begin{array}{*{20}c} {\text{if}} & {x \ge 0} \\ \end{array} } \\ 0 & {\begin{array}{*{20}c} {\text{if}} & {x < 0} \\ \end{array} .} \\ \end{array} } \right.$$

where the slope *m* is unknown.

In accordance with (30), it can be inferred that if $h\left( {x,\alpha } \right) \cong mx$, then $\frac{{h\left( {x,\alpha } \right)}}{mx} \cong 1$. As the same occurs with $h\left( { - x,\alpha } \right)$ (that is, $h\left( { - x,\alpha } \right) \cong - mx$ and $\frac{{h\left( { - x,\alpha } \right)}}{ - mx} \cong 1$), we consider in this proof only $h\left( {x,\alpha } \right)$ since the final result will remain also valid for $h\left( { - x,\alpha } \right)$.

Consequently,

31 $$\frac{{h\left( {x,\alpha } \right)}}{mx} \cong \frac{{\ln \left( {1 + e^{\alpha x} } \right)}}{{\alpha \ln \left( {10} \right)mx}} \cong \frac{1}{{m\ln \left( {10} \right)}}\frac{{\ln \left( {1 + e^{{\left( {\alpha x} \right)}} } \right)}}{{\left( {\alpha x} \right)}} \cong 1$$

After a change of variable $X = \alpha x$, from (31) we have

32 $$\frac{h(x,\alpha )}{mx} \cong \frac{1}{m\ln (10)}\frac{{\ln \left( {1 + e^{(X)} } \right)}}{(X)} \cong 1$$

Hence, taking into account that the approximations of (30) are completely valid for huge values of *α* and $x$, then an estimated value of $\frac{{\ln (1 + e^{(X)} )}}{(X)}$ can be obtained by considering the following limit evaluation:

33 $$\begin{array}{*{20}c} {\lim } \\ {X \to + \infty } \\ \end{array} \frac{{\ln \left( {1 + e^{(X)} } \right)}}{(X)} \cong 1$$

From this result, Eq. (32) can be simplified as

34 $$\frac{h(x,\alpha )}{mx} \cong \frac{1}{m\ln (10)} \cong 1$$

where $m$ is given by

35 $$m \cong \frac{1}{\ln (10)}$$

whose value deviates from the unity slope presented in the absolute-value function of reference ($\left| x \right|$). From here, in order to force an unity slope in both, $h(x,\alpha )$ (for $x \ge 0$) and $h( - x,\alpha )$ (for $x \le 0$), a compensation constant $\mu = \ln (10)$ is included in (29) as

36 $$\hat{h}(x,\alpha ) = \mu h(x,\alpha ) = \frac{\mu }{\alpha \ln (10)}\ln \left( {1 + e^{\alpha x} } \right) = \frac{1}{\alpha }\ln \left( {1 + e^{\alpha x} } \right)$$

which allows recast the approximation (3) in terms of $\hat{h}(x,\alpha )$ and $\hat{h}( - x,\alpha )$ as follows:

37 $$\left| x \right| \cong \hat{h}(x,\alpha ) + \hat{h}( - x,\alpha ) \cong \frac{1}{\alpha }\left[ {\ln \left( {1 + e^{ - \alpha x} } \right) + \ln \left( {1 + e^{{ {\alpha x} }} } \right)} \right]$$

Finally, after algebraic simplifications of (37), it yields

38 $$\left| x \right| = \frac{2}{\alpha }\ln \left( {e^{{\left( {\frac{\alpha }{2}x} \right)}} + e^{{\left( {\frac{ - \alpha }{2}x} \right)}} } \right)$$

## Appendix B

Consider that the canonical piecewise-linear function $y_{cpwl} (x)$ and its smooth-piecewise version $y_{s} (x)$ have the form

39 $$y_{cpwl} (x) = a + bx + \sum\limits_{i = 1}^{\sigma } {c_{i} \left| {x - \beta_{i} } \right|}$$

and

40 $$y_{s} (x) = \left( {a - \sum\limits_{i = 1}^{\sigma } {c_{i} \beta_{i} } } \right) + \left( {b + \sum\limits_{i = 1}^{\sigma } {c_{i} } } \right)x + \sum\limits_{i = 1}^{\sigma } {\left( {\frac{{2c_{i} }}{\alpha }} \right)\ln \left( {1 + e^{{ - \alpha (x - \beta_{i} )}} } \right)}$$

Suppose that we do not like that the approximation $y{}_{s}(x)$ differs from the original function $y_{cpwl} (x)$ too much around to any specific breakpoint $\beta_{i}$.

That is

41 $$\left| {y_{cpwl} (\beta_{i} ) - y_{s} (\beta_{i} )} \right| < \delta$$

After substituting $x = \beta_{i}$ in (39) and (40), the evaluation of (41) yields

42 $$\left| { - \frac{{2c_{i} \ln (2)}}{{\alpha_{i} }}} \right| < \delta \quad {\text{or}}\; \frac{{2c_{i} \ln (2)}}{{\alpha_{i} }} < \delta$$

where a value of the smoothing parameter $\alpha_{i}$ that ensures fulfilling the condition (41) is given by

43 $$\alpha_{i} > \frac{{2c_{i} \ln (2)}}{\delta }$$

with $\alpha_{i}$ being a positive number, and more generally, for a specific deviation $\delta$, it yields

44 $$\alpha_{i} = \frac{{2c_{i} \ln (2)}}{\delta }\quad {\text{for}}\;i = 1 \ldots \sigma$$

## Appendix C

Firstly, consider the *n*-dimensional form of (6) given by

45 $$\left| {\mathbf{x}} \right| \cong \left( {\frac{2}{\alpha }} \right)\ln \left( {e^{{\frac{\alpha }{2}\left( {\left\langle {{\user2{\Lambda}}^{\left( i \right)} ,{\mathbf{x}}} \right\rangle - \beta_{i} } \right)}} + e^{{\frac{ - \alpha }{2}\left( {\left\langle {{\user2{\Lambda}}^{\left( i \right)} ,{\mathbf{x}}} \right\rangle - \beta_{i} } \right)}} } \right)$$

then, after replacing the form of each absolute-value term of (2) by its smooth approximation (45) results

46 $$y\left( {\mathbf{x}} \right) = a + {\mathbf{{\rm B}x}} + \sum\limits_{i = 1}^{\sigma } {\left( {\frac{{2c_{i} }}{\alpha }} \right)\ln \left( {e^{{\frac{\alpha }{2}\left( {\left\langle {{\user2{\Lambda}}^{(i)} ,{\mathbf{x}}} \right\rangle - \beta_{i} } \right)}} + e^{{\frac{ - \alpha }{2}\left( {\left\langle {{\user2{\Lambda}}^{(i)} ,{\mathbf{x}}} \right\rangle - \beta_{i} } \right)}} } \right)}$$

for $$i = 1, \ldots ,\sigma$$

with ${\mathbf{x}} = \left[ {x_{1} , \ldots ,x_{n} } \right]$ expressed as $(1 \times n)$ matrix, and both, ${\mathbf{\rm B}} = \left[ {b_{1} , \ldots ,b_{n} } \right]^{T}$ and ${\user2{\Lambda}}^{(i)} = \left[ {\lambda_{1}^{(i)} , \ldots ,\lambda_{n}^{(i)} } \right]^{T}$ expressed as $(n \times 1)$ matrices.

After that, an algebraic expansion of the logarithm argument in (46) yields

47 $$y({\mathbf{x}}) = a + {\mathbf{{\rm B}x}} + \sum\limits_{i = 1}^{\sigma } {\left( {\frac{{2c_{i} }}{\alpha }} \right)\ln \left( {\frac{{1 + e^{{ - \alpha \left( {\left\langle {{\user2{\Lambda}}^{(i)} ,{\mathbf{x}}} \right\rangle - \beta_{i} } \right)}} }}{{e^{{\frac{ - \alpha }{2}\left( {\left\langle {{\user2{\Lambda}}^{(i)} ,{\mathbf{x}}} \right\rangle - \beta_{i} } \right)}} }}} \right)}$$

using the property $\ln \left( {\frac{u}{v}} \right) = \left( {\ln (u) - \ln (v)} \right)$ and simplifying we have

48 $$y({\mathbf{x}}) = a + {\mathbf{{\rm B}x}} + \sum\limits_{i = 1}^{\sigma } {c_{i} \left( {\left\langle {{\user2{\Lambda}}^{(i)} ,{\mathbf{x}}} \right\rangle - \beta_{i} } \right) + \sum\limits_{i = 1}^{\sigma } {\left( {\frac{{2c_{i} }}{\alpha }} \right)\ln \left( {1 + e^{{ - \alpha \left( {\left\langle {{\user2{\Lambda}}^{(i)} ,{\mathbf{x}}} \right\rangle - \beta_{i} } \right)}} } \right)} }$$

In order to simplify (48) we collect like terms

49 $$y({\mathbf{x}}) = \left( {a - \sum\limits_{{i = 1}}^{\sigma } {c_{i} \beta _{i} } } \right) + \left( {{\mathbf{B + }}\sum\limits_{{i = 1}}^{\sigma } {c_{i} {\mathbf{\Lambda }}^{{(i)}} } } \right){\text{x + }}\sum\limits_{{i = 1}}^{\sigma } {\left( {\frac{{2c_{i} }}{\alpha }} \right)\ln \left( {1 + e^{{ - \alpha \left( {\left\langle {\Lambda ^{{(i)}} ,{\text{x}}} \right\rangle - \beta _{i} } \right)}} } \right)}$$

finally, in accordance to the form of (14), we obtain

$$A = a - \sum\limits_{i = 1}^{\sigma } {c_{i} \beta_{i} }$$$${{\hat{\rm B}}} = {{\rm B}} + \sum\limits_{i = 1}^{\sigma } {c_{i} {\user2{\Lambda}}^{(i)} }$$$$C_{i} = \frac{{2c_{i} }}{\alpha }$$ for $$i = 1 \ldots \sigma$$
